# The Use of Ipratropium Bromide for the Treatment of Pediatric Sialorrhea: A Retrospective Clinical Case Series

**DOI:** 10.1177/00034894241235523

**Published:** 2024-03-03

**Authors:** Suhaima Tunio, Julie E. Strychowsky, Agnieszka Dzioba, Peng You, Edward Madou, Breanna A. Chen

**Affiliations:** 1 Schulich School of Medicine & Dentistry, Western University, London, ON, Canada; 2 Department of Otolaryngology-Head & Neck Surgery, Schulich School of Medicine & Dentistry, Western University, London, ON, Canada; 3 Department of Paediatrics, Schulich School of Medicine & Dentistry, Western University, London, ON, Canada; 4 Children’s Health Research Institute, Lawson Health Research Institute, London, ON, Canada

**Keywords:** sialorrhea, ipratropium bromide, anticholinergic agents, case reports, drooling

## Abstract

**Objective:**

This retrospective review documents the experience of ipratropium bromide use among pediatric patients with sialorrhea at our multidisciplinary sialorrhea clinic at Children’s Hospital at London Health Sciences Centre (LHSC).

**Methods:**

A retrospective chart review of patients diagnosed with sialorrhea at our multidisciplinary clinic between January 2015 and June 2021 was completed. Data on patient demographics, comorbidities, clinical presentation, previous interventions, quality of life, and medication adverse side effects was collected. Drooling Frequency and Severity Scale (DFSS) scores were reviewed to compare sialorrhea management pre- and post-treatment with topical 0.03% ipratropium bromide nasal solution. A descriptive analysis and Wilcoxon signed rank tests were conducted to compare pre- versus post-treatment DFSS scores.

**Results:**

A total of 12 patients presented for follow-up and were included in the final analysis. At the pre-treatment visit, the median DFSS score was 4 for frequency and 5 for severity. Post-treatment, median DFSS score was 3 for frequency and 4.5 for severity, (*P* = .020 and .129, respectively). Minimal adverse effects were encountered.

**Conclusions:**

Ipratropium bromide provided a statistically significant benefit for drooling frequency in the patients studied and may present an additional topical medical option for pediatric sialorrhea with limited adverse effects.

## Introduction

Sialorrhea is a chronic problem seen in pediatric patients with oral-motor dysfunction.^1^ The prevalence of sialorrhea varies based on underlying comorbidities. In patients with cerebral palsy, the prevalence of sialorrhea ranges from 10% to 58%.^2^ Sequelae of sialorrhea include aspiration pneumonia, dermatologic problems (facial irritation and skin excoriation), and damage to dentition.^3^ Furthermore, sialorrhea can have negative impacts on social interactions and treatment for sialorrhea has been reported to increase self-esteem and social contacts in patients with cerebral palsy.^3^

Both medical and surgical options for sialorrhea treatment exist. Surgical options aim to reduce saliva production, redirect salivary flow, or a combination of both. These interventions include intraglandular injection of botulinum toxin^4,5^ and salivary gland excision or duct ligation.^6,7^ The overall success rate for various surgical procedures for the treatment of sialorrhea is reported to be between 31% and 86%.^8^ However, recurrence is reported to occur in 68% of patients, with additional therapies being required for a significant number of patients following surgical treatment.^8^

In comparison, medical therapy for sialorrhea predominantly targets the parasympathetic system to reduce saliva production. Physiologically, salivation occurs due to the stimulation of muscarinic subtype 3 receptors, and it is mediated by the parasympathetic innervation of salivary glands. By blocking cholinergic muscarinic receptors, anticholinergic medications act as antisialogogues.^9^

Anticholinergic agents used in sialorrhea management include sublingual drops (atropine),^10,11^ oral tablets or suspensions (benztropine^12^ and glycopyrrolate^13,14^), transdermal patches (scopolamine/hyoscine^15^), and sublingual application of sprays (ipratropium bromide^16–18^). By acting on the parasympathetic system, systemic anticholinergic medications may cause several adverse side effects such relaxation of bowel smooth muscle and the detrusor muscle of the bladder causing constipation and urinary retention, flushing, tachycardia, and accommodative issues causing blurred vision.^19^ Anticholinergics also have nervous system mediated side effects such as irritability, sedation, headache, and more frequent seizures.^19^ There is a lack of consensus on which pharmaceutical agent is most efficacious for sialorrhea.^20^

One frequently used medication in our center to control sialorrhea is atropine ophthalmic drops, delivered sublingually. An alternative to atropine ophthalmic drops has been necessary due to regional atropine drug shortages. Ipratropium bromide is an anticholinergic that is structurally similar to atropine and has nonspecific muscarinic receptor blockage with minimal effects on nicotinic receptors as well as no central nervous system penetration. As such, ipratropium bromide has been utilized in pediatric patients at the Children’s Hospital at London Health Sciences Centre (LHSC) multidisciplinary sialorrhea clinic. There is currently a paucity of research on the use and efficacy of ipratropium bromide for sialorrhea in pediatric patients.

The purpose of this retrospective review is to document the experience of ipratropium bromide use among pediatric patients with sialorrhea at the multidisciplinary sialorrhea clinic at LHSC.

## Methods

### Data Collection

A retrospective chart review of pediatric patients diagnosed with sialorrhea seen at our multidisciplinary clinic between January 2015 and June 2021 was completed. The multidisciplinary clinic is staffed by a pediatric otolaryngologist and a pediatrician. Patients were included in the review if they were treated with ipratropium bromide for sialorrhea during the study period and excluded if a follow-up assessment was not available. Patients were prescribed 1 to 2 sprays of 21 µg (0.03%) ipratropium bromide nasal spray administered sublingually or buccally up to 3 times a day as needed. Local institutional Review Board approval was obtained from Western University Research Ethics Board (REB# 119482).

Study data were collected and managed using REDCap electronic data capture tools. Patient demographics, comorbidities, clinical presentation, previous and concurrent interventions, drooling frequency, severity questionnaires, and medication adverse side effects were collected.

**Figure 1.**
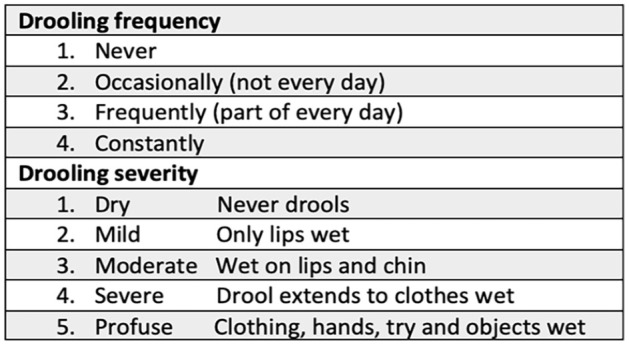
Drooling frequency and severity scale.

The primary study outcome, efficacy of ipratropium bromide for treatment of sialorrhea, was evaluated via subjective caregiver appraisals of drooling frequency and severity, using the Drooling Frequency and Severity Scale (DFSS) developed by Thomas-Stonell and Greenberg (Figure 1).^21^ DFSS scores were collected during patient appointments and were reviewed to compare sialorrhea management pre- and post-treatment.

### Data Analysis

A descriptive analysis of the clinical profile and treatment outcomes of the study population was undertaken. Summary statistics, including medians with respective interquartile ranges, and frequency statistics, were calculated for the appropriate variable types. Wilcoxon signed rank tests were then conducted to compare pre- versus post-treatment DFSS scores. Statistical analyses were undertaken using SPSS (IBM Corp. Released 2017. IBM SPSS Statistics for Windows, Version 27.0. Armonk, NY: IBM Corp). Statistical significance was set a priori at an alpha of .05. Moreover, a 1-point difference in DFSS scores was deemed to be clinically significant.

## Results

Twenty-six patients were treated with ipratropium bromide from 2015 to 2021. Of these, 12 patients presented for follow-up appointments and were included in the final analysis.

### Patient Characteristics

The median age at initial treatment was 7.2 years [interquartile range (IR), 3.7 years] and 50% of the patients were male. The most common primary diagnoses were confirmed chromosomal abnormality in 50% and cerebral palsy in 25% of the patients. All had developmental delays with 10/12 patients diagnosed with global developmental delay, 1 patient with motor and speech delays and the remaining patient with a diagnosis of autism spectrum disorder. About 50% of patients had a comorbid diagnosis of epilepsy. Table 1 characterizes patient characteristics.

**Table 1.** Patient Characteristics and Pre- and Post-Ipratropium Bromide DFSS Scores.

Patient number	Age (years)	Gender	Primary diagnoses	Comorbidities	Previous treatments	Pre DFSS	Post DFSS
1	13	M	Angelman syndrome and GDD	Epilepsy, vision loss, and constipation	None	4,4	3,4
2	9	M	Speech and motor delays and learning disability	Allergic rhinitis and asthma	None	4,3	3,3
3	5	F	Cask-related disorder, dystonic cerebral palsy, and GDD	Hearing loss, vision loss, asthma, constipation, and recurrent UTIs	None	4,5	3,5
4	3	F	Genetic mutation IQSEC 2 and GDD	Epilepsy and hypotonia	None	4,4	2,2
5	5	M	Spastic cerebral palsy, GDD, and extreme prematurity	Tracheostomy dependence, vocal cord dysfunction, and chronic lung disease	Atropine	4,5	4,5
6	6	F	SCNA2A mutation and GDD	Epilepsy, hypotonia, and vision loss	None	3,4	3,5
7	4	M	Agenesis corpus callosum and GDD	Dysplastic right kidney	None	4,5	3,4
8	6	F	Unbalanced translocation of chromosome 7/11 and GDD	ASD, microcephaly, and vision loss	None	4,5	4,5
9	6	M	Autism spectrum disorder	Query asthma	None	4,5	3,3
10	10	F	Chromosome 3p duplication and GDD	Epilepsy and celiac disease	Botox, atropine, glycopyrrolate	4,5	4,5
11	11	M	Cerebral palsy and GDD	Epilepsy	4 gland ligations, Botox, atropine, and glycopyrrolate	4,5	4,5
12	1	F	Suspected genetic disorder and GDD	Epilepsy and neurogastrointestinal dysmotility	Atropine	3,4	3,3

Abbreviations: ASD, autism spectrum disorder; GDD, global developmental delay.

### Sialorrhea Treatment

Four patients had previously trialed either pharmacologic or surgical treatments (Table 1). All 4 received atropine, 2 (50%) received glycopyrrolate, 2 (50%) patients received botulinum toxin injections to the salivary glands, and 1 underwent a 4-gland salivary duct ligation prior to the decision to trial ipratropium bromide. No patient confirmed using concurrent sialorrhea therapies while taking ipratropium bromide, but 1 patient had concurrent prescriptions for both ipratropium bromide and glycopyrrolate with the intent to titrate use of both.

The patients in this study reported using a range of 1 to 2 sprays 1 to 3 times daily. The duration of usage was clear for only 5/12 of the patients and ranged from <1 week to more than 2 years. Three patients (25%) continued to use ipratropium bromide at the conclusion of the study or until discharge from the clinic while 9 (75%) patients had discontinued or chose to discontinue medication at follow-up. Reasons for discontinuation included lack of perceived efficacy (7/9; 78%), difficulty with administration (3/9; 33%), and adverse xerostomia effect (1/9; 11%). Of the patients who discontinued, 3 (33%) consented for botulinum toxin injections, and 5 (56%) pursued atropine treatment.

### Efficacy of Ipratropium Bromide

**Figure 2.**
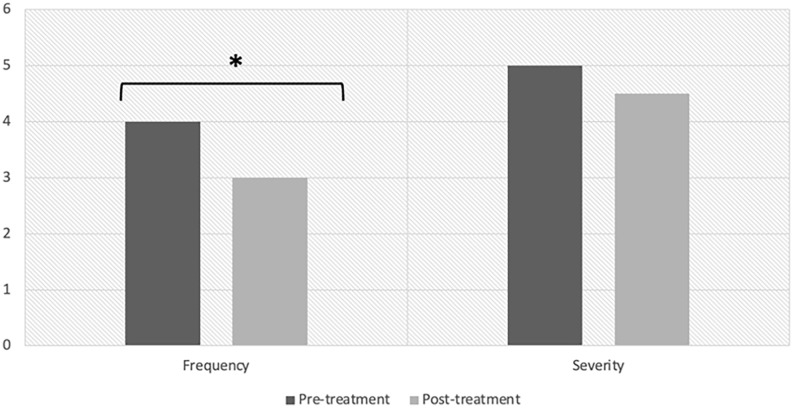
DFSS Scores pre- and post-ipratropium bromide treatment. The results of this study demonstrated a statistically significant reduction in drooling frequency. (*p = 0.020).

At the pre-treatment visit, the median DFSS score was 4 and 5 for frequency and severity, respectively. The median score improved to a frequency and severity of 3 and 4.5, respectively, at the post follow-up visit (*P* = .020 and .129, respectively), achieving a statistically significant improvement in drooling frequency. Results are displayed in Figure 2. This corresponds with a change in frequency from “constantly drools” to “frequently drools” and a change in severity from “profuse drooling” to “severe drooling,” which we considered a priori to reflect a clinically significant improvement (Figure 1). Regarding adverse effects or patient concerns, 2 patients’ caregivers reported difficulty administering the spray due to taste intolerance, and 1 patient reported excessively dry or thick secretions in the setting of tracheostomy. Table 1 summarizes the patient characteristics and the pre- and post- ipratropium bromide DFSS scores of the patients in this study.

## Discussion

While anticholinergic medications serve as the primary medical therapy option for sialorrhea, differences in efficacy, administration, side effect profiles, and cost help inform choice of medication. Glycopyrrolate is the most studied in the pediatric population and has been shown to be an effective medication for sialorrhea.^18^ Regional access to glycopyrrolate may be challenging due to cost. For instance, glycopyrrolate is not covered by the Ontario publicly-funded drug program. Scopolamine patches are also described in the literature ^18^ but can be difficult to titrate, have been associated with more reported systemic side effects^14^ and are discontinued from the Canadian market as of 2022. Atropine ophthalmic drops, administered sublingually, are a commonly prescribed option in our clinic, however temporary medication shortages necessitated a practical alternative.

Ipratropium bromide spray, administered sublingually or buccally, provided a statistically significant benefit for drooling frequency. A priori, we had forecast a change in DFSS score of 1 point to be clinically significant, in this case, decreasing from “constantly” drooling to “frequently” drooling. However, having 7/12 of our patients discontinue the medication due to lack of efficacy warrants consideration as well. There was no statistically significant change in drooling severity. It remains to be determined whether this overall decrease in scores is clinically significant or not.

The reduction in subjective salivation scores following ipratropium bromide seen in our study have been reported in other patient populations. Ipratropium bromide has been shown to reduce subjective appraisals of salivation frequency in patients with Parkinson’s disease and clozapine-induced hypersalivation.^15,16^ However, in Parkinson’s disease, the same effect was found following the use of a matching placebo,^16^ therefore the efficacy remains inconclusive.

Similar to previous studies, our retrospective case series illustrates that ipratropium bromide is generally well tolerated and has minimal side effects.^15,16^ Within our patient cohort, the only reported side effect from ipratropium bromide was excessively thick secretions in the setting of tracheostomy. Some parents reported difficulty administering the spray due to the patients not tolerating the taste of the medication, which can pose a difficulty in the pediatric population.

There are several limitations to this study that limit the scientific interpretation. Firstly, the study population of 12 patients is small and heterogeneous in underlying primary diagnoses. The retrospective study design is reliant on the availability of adequate documentation and precludes the use of a placebo control for comparison. Loss to follow up was significant, with only 12/26 patients prescribed ipratropium bromide present for follow up appointments. The latter half of the study period took place during the COVID-19 pandemic, and lack of follow-up may have been secondary to patient and caregiver preference to avoid hospital clinic visits in addition to administrative limitations on clinical volumes. There was considerable heterogeneity in the usage and administration of medication and much of the data regarding length of ipratropium bromide use was missing. A prolonged duration until post-treatment scores were collected may have also been affected by developmental progress and thus natural decline in DFSS scores over time. Furthermore, the DFSS scale used in this study is a subjective scale scored by parents and is not validated in the literature. Given these limitations, additional research with more consistent medication administration and validated outcome measures is warranted to further understand the utility of ipratropium bromide in this patient population.

In conclusion, ipratropium bromide 0.03% nasal solution may present an additional topical medical option for pediatric sialorrhea with limited adverse effects. This retrospective review of our experience indicates a significant improvement in drooling frequency, however, heterogeneity in the severity of drooling and heterogeneity in the usage and administration of medication make the scientific interpretation limited. Future studies are needed to further explore the impact of ipratropium bromide on the treatment of sialorrhea in pediatric patients.

## References

[1] Hussein I Kershaw AE Tahmassebi JF Fayle SA. The management of drooling in children and patients with mental and physical disabilities: a literature review. Int J Paediatr Dent. 1998;8(1):3-11. doi:10.1046/j.1365-263x.1998.00055.x 9558540

[2] Dias BLS Fernandes AR Maia HS Filho . Treatment of drooling with sublingual atropine sulfate in children and adolescents with cerebral palsy. Arq Neuropsiquiatr. 2017;75(5):282-287. doi:10.1590/0004-282X20170033 28591387

[3] van der Burg JJ Jongerius PH van Limbeek J van Hulst K Rotteveel JJ. Social interaction and self-esteem of children with cerebral palsy after treatment for severe drooling. Eur J Pediatr. 2006;165(1):37-41. doi:10.1007/s00431-005-1759-z 16172877

[4] Jongerius PH van den Hoogen FJ van Limbeek J Gabreëls FJ van Hulst K Rotteveel JJ. Effect of botulinum toxin in the treatment of drooling: a controlled clinical trial. Pediatrics. 2004; 114(3):620-627. doi:10.1542/peds.2003-1104-L 15342830

[5] Lagalla G Millevolte M Capecci M Provinciali L Ceravolo MG. Botulinum toxin type A for drooling in Parkinson’s disease: a double-blind, randomized, placebo-controlled study. Mov Disord. 2006;21(5):704-707. doi:10.1002/mds.20793 16440332

[6] Reed J Mans CK Brietzke SE. Surgical management of drooling: a meta-analysis. Arch Otolaryngol Head Neck Surg. 2009;135(9):924-9431. doi:10.1001/archoto.2009.110 19770427

[7] Khan WU Islam A Fu A , et al. Four-duct ligation for the treatment of sialorrhea in children. JAMA Otolaryngol Head Neck Surg. 2016;142(3):278-283. doi:10.1001/jamaoto.2015.3592. PMID: 26869013 26869013 10.1001/jamaoto.2015.3592

[8] Martin TJ Conley SF. Long-term efficacy of intra-oral surgery for sialorrhea. Otolaryngol Head Neck Surg. 2007;137(1):54-58. doi:10.1016/j.otohns.2007.01.034 17599565

[9] Proctor GB. The physiology of salivary secretion. Periodontol 2000. 2016;70(1):11-25. doi:10.1111/prd.12116 26662479

[10] Rapoport A. Sublingual atropine drops for the treatment of pediatric sialorrhea. J Pain Symptom Manage. 2010;40(5):783-788. doi:10.1016/j.jpainsymman.2010.02.007 20541902

[11] Norderyd J Graf J Marcusson A , et al. Sublingual administration of atropine eyedrops in children with excessive drooling – a pilot study. Int J Paediatr Dent. 2017;27(1):22-29. doi:10.1111/ipd.12219 26708211 PMC5324542

[12] Camp-Bruno JA Winsberg BG Green-Parsons AR Abrams JP. Efficacy of benztropine therapy for drooling. Dev Med Child Neurol. 1989;31(3):309-319. doi:10.1111/j.1469-8749.1989.tb04000.x 2666205

[13] Garnock-Jones KP. Glycopyrrolate oral solution: for chronic, severe drooling in pediatric patients with neurologic conditions. Paediatr Drugs. 2012;14(4):263-269. doi:10.2165/11208120-000000000-00000 22646067

[14] Mier RJ Bachrach SJ Lakin RC Barker T Childs J Moran M. Treatment of sialorrhea with glycopyrrolate: A double-blind, dose-ranging study. Arch Pediatr Adolesc Med. 2000;154(12):1214-1218. doi:10.1001/archpedi.154.12.1214 11115305

[15] Lewis DW Fontana C Mehallick LK Everett Y. Transdermal scopolamine for reduction of drooling in developmentally delayed children. Dev Med Child Neurol. 1994;36(6):484-486. doi:10.1111/j.1469-8749.1994.tb11877.x 7516297

[16] Calderon J Rubin E Sobota WL. Potential use of ipatropium bromide for the treatment of clozapine-induced hypersalivation: a preliminary report. Int Clin Psychopharmacol. 2000;15(1):49-52. doi:10.1097/00004850-200015010-00008 10836287

[bibr17-00034894241235523] Thomsen TR Galpern WR Asante A Arenovich T Fox SH. Ipratropium bromide spray as treatment for sialorrhea in Parkinson’s disease. Mov Disord. 2007;15;22(15):2268-2273. doi:10.1002/mds.21730 17876852

[18] Freudenreich O Beebe M Goff DC. Clozapine-induced sialorrhea treated with sublingual ipratropium spray: a case series. J Clin Psychopharmacol. 2004;24(1):98-100. doi:10.1097/01.jcp.0000106228.36344.2e 14709958

[19] Mier RJ Bachrach SJ Lakin RC Barker T Childs J Moran M. Treatment of sialorrhea with glycopyrrolate: A double-blind, dose-ranging study. Arch Pediatr Adolesc Med. 2000;154(12):1214-1218. doi:10.1001/archpedi.154.12.1214 11115305

[20] You P Strychowsky J Gandhi K Chen BA. Anticholinergic treatment for sialorrhea in children: a systematic review. Paediatr Child Health. 2021;27(2):82-87. doi:10.1093/pch/pxab051 35599670 PMC9113838

[21] Thomas-Stonell N Greenberg J. Three treatment approaches and clinical factors in the reduction of drooling. Dysphagia. 1988;3(2):73-78. doi:10.1007/BF02412423 3271655

